# Development of Language and Pragmatic Communication Skills in Preschool Children with Developmental Language Disorder in a Speech Therapy Kindergarten—A Real-World Study

**DOI:** 10.3390/children12070921

**Published:** 2025-07-11

**Authors:** Dieter Ullrich, Magret Marten

**Affiliations:** 1 Medical Practice Pediatric/ENT, Wedemarkstr. 83, 30900 Wedemark, Germany; 2 Logopaedic Kindergarten, An der Autobahn 2, 30851 Langenhagen, Germany; magretmarten@gmx.de

**Keywords:** verbal communication development, preschool children with speech-language disorder, communicative participation, logopaedic kindergarten, importance of SL-therapy and educational measures

## Abstract

Background: Several studies document the importance of communicative abilities for children’s development. Especially in recent years verbal communication in preschool children with developmental language disorder (DLD) has been studied, relying heavily on statistical analysis, outcome measures, or/and parents’ reports. Purpose: This explorative study investigates the effects of speech therapy on the development of language and verbal communication skills in preschool children with DLD within their peer group in a day-to-day setting using objective video-documentation. Hypothesis: Speech therapy leads to improvement of language, communication, and possibly to concurrent development of both language and verbal communication skills in preschool children. Methods: Preliminary prospective study to assess language and verbal communications skills of nine preschool children (seven boys, two girls, 4–6 y) with DLD in a speech therapy kindergarten using video recordings over a one-year therapy period. The communicative participation of the members of the peer group was assessed and included the verbal address (Av) and the ratio of “verbal address/verbal reaction” (Av/Rv). Results: The investigation results in evidence for two outcome groups: One group with suspected preferential verbal communication disorders (*n* = 4) was characterised by a high Av/Rv value, meaning they were scored to have a normal or high verbal address (Av) and a low verbal response (Rv) (predominantly interpersonal communication related disorder). This group showed minimal changes in the short term but demonstrated improvement after 5 years of schooling; thus, pedagogical activities seemed to be particularly effective for these children. The second group showed a balanced Av/Rv ratio (predominantly language related disorder) (n = 5); but after five years they demonstrated a partial need for special school support measures. This group may therefore particularly benefit from speech therapy. Conclusions: The present study clearly shows that even with speech-language therapy, the linguistic ability of DLD-disturbed children does not necessarily develop simultaneously with their communication ability. Rather, the investigations provide evidence for two groups of preschool children with DLD and communication disorder: One group demonstrated a predominantly verbal communication related disorder, where pedagogical intervention might be the more important treatment. The second group showed predominantly DLD, therefore making speech therapy the more effective intervention. In this study, all children expressed their desire to communicate with their peers. To the authors’ best knowledge, this is the first study determining the ability to communicate in a preschool cohort with DLD using characterisation with video documentation in a follow-up for 1 year.

## 1. Introduction

Human communication involves the interpersonal exchange of ideas, wishes, and feelings among people [1,2,3]. The development of social and verbal communication skills is a significant developmental step for infants and children and is critical for their intelligence, the development of cognition, their future communicative participation, and thus for their future lives [2,4]. Earlier research has primarily investigated children’s speech and language skills, but in recent years the importance of pragmatic communicative skills has been recognised [1,2,3,4,5,6,7,8]. The scientific and educational importance of communication skills is reflected in the introduction of social (pragmatic) communication disorder as a new category in the fifth edition of the *Diagnostic and Statistical Manual of Mental Disorders* [9]. The importance of communication is also emphasised in the International Classification of Functioning, Disability and Health (ICF) [10]. Nevertheless, uncertainties and unanswered questions remain [11].

The concept of communication is complex. An infant’s smiling and crying represent the first type of communication [12]. Andalo et al. [13] documented that the development of language and communication is partially based on physical motoric development. According to Piaget et al. [14] and Zollinger [15], the development of communication is linked to the child’s ability to play, recognise symbols, and triangulate. In recent years, numerous publications and reviews on child communication have appeared and contributed to a better understanding [6,16,17,18,19]. However, it should not be overlooked that individual elements of communication do not necessarily reflect communicative participation. Ultimately, however, it is communicative participation that is of crucial importance [7,11].

To assess communication skills in preschool children, some studies attempted caregiver questionnaires. The FOCUS (*Focus on the Outcomes of Communication Under Six*) questionnaire examines the communicative skills of subjects under the age of six years and is also used to correlate communication and DLD development in children [20]. In the vast majority of all studies, the development of communication is assessed on the analysis of various test procedures or questionnaires and, furthermore, on patient-reported outcome measures or parents’ reports concerning communicative skills [16]. These approaches often only provide indirect (evaluative) and/or retrospective information regarding communication skills. However, communicative skills do not necessarily equate to possessing the ability for “communicative participation” [7,11,18]. To our best knowledge currently there exist no objective testing methods to characterise communicative abilities in preschool children.

In Germany, specialised logopaedic/speech therapy kindergartens exist. The concept of logopaedic kindergartens is founded on scientific evidence showing that children with speech and language impairments benefit from appropriate therapy. The multimodal approach of these kindergartens involving the participation of specialised educators, speech therapists, occupational therapists, and psychologists aims to help the children to integrate into mainstream schools and pursue vocational training later in life [21,22].

Prior to admission to a speech therapy kindergarten, other significant impairments apart from speech development disorders must be excluded. Furthermore, children must have received 30 h or more of unsuccessful outpatient speech therapy. Upon admission, children are aged between 4 and 6 years, and the average duration of therapy is 18 months. The ratio of children to educators in the logopaedic kindergarten is 8:1.5, thus significantly better than that in regular kindergarten with a ratio of 25:3.

Pedagogical research [23,24] has shown that objective skills such as a high IQ (hard skill) do not necessarily guarantee success or stimulate optimal development. Instead, soft skills, such as the environment or intensive practice (life skills), seem to be crucial for a positive long-term development. The authors suggest that this situation might be comparable for speech-language development. This implies that, in agreement with Cunningham et al. [7,18], individual language skills, such as articulation, vocabulary, or phonological awareness (hard skills) or single communicative skills alone are not sufficient for children’s language or communicative development and participation outcome. It is important that soft skills, such as interindividual communication and language practice, must be further developed. Enhancing these soft skills could potentially improve the long-term prognosis of children with DLD and communication disorders.

Consequently, this explorative study investigated verbal interpersonal communication of preschool children with their peers as an important aspect of communicative participation behaviour in young children with DLD. Additionally, we introduced video analysis as a novel method to analyse communicative development and participation in preschool children. One important research hypothesis investigates whether speech-language therapy will improve DLD and communication concurrently.

## 2. Methods

### 2.1. Study Design

The authors present a prospective observational study with preschool children in a speech therapy kindergarten in Hanover, Germany, over one therapy year (2016 to 2017). As part of the admission process, parents were required to consent to various examinations, possible scientific analyses, and publications. This informed consent was obtained from parents and caregivers prior to enrolment. No additional examinations were conducted specifically for this study.

### 2.2. Participants

Nine preschool children (7 boys, 2 girls) (Table 1) with DLD were included in the study. At study enrolment, the children were aged 4–6 years. All children had a pronounced developmental language disorder (ICD-10: F 80.9) and minor additional disorders, such as problems associated with the oral musculature (ICD-10: F 82) [25] (see Appendix A). Table 1 summarises the characteristics of the study participants, including age, native family language, IQ, number of siblings, and characterisation of SL-disorder according to ICD [25]. Five children came from non-native German-speaking families (Child: D1, P, D2, J1, E). However, German was also the everyday language in these households. Logopaedic speech therapy assessment established that the extent of German language problems was comparable in all study participants, regardless of their language background (Appendix A). For children of a non-German-speaking background, caregiver feedback confirmed the similarities of speech problems in both languages. The details on the different logopaedic criteria and the language status before and after therapy are presented in Appendix A.

All children underwent a psychological and a speech-therapy assessment upon admission. Within the therapy year all children were routinely tested 4 times per therapy year with regard to their language skills and 2 to 3 times per year regarding their overall development. Similar to the initial assessments, these examinations are based on various validated test procedures (Table 2), which were individually conducted by experienced examiners under optimal conditions. After evaluation and additional consideration of the individual assessments by educators and therapists (Table 2 and Table 3; Appendix A, Appendix B, Appendix C and Appendix D), the further logopaedic and pedagogical treatment concept was developed and adapted.

The assessment data on the children’s various abilities and characteristics and the changes in some of these during the therapy year are shown in condensed form in Table 3 and in Appendix A, Appendix B, Appendix C and Appendix D.

### 2.3. Methods and Analysis of Communication Skills

The authors developed an internal assessment protocol for communication evaluation previously unreported.

Based on this protocol, the children’s communication behaviour was assessed qualitatively or semi-quantitatively by educators and speech therapists using standardised methodology on admission and at regular intervals of 3 to 4 months (Table 2 and Table 3; Appendix A and Appendix B).

Given the children’s frequent difficulties in passing objective tests, all children in the study groups were also routinely assessed using Zollinger’s developmental profiles (Appendix B). Zollinger’s [15] non-standardised developmental profiles are qualitative assessments of four skills (i.e., practical-gnostic, symbolic, linguistic, social-communicative criteria) which have shown to be present in approximately 80% of children aged 9 to 42 months. However, Zollinger’s test results in the present study are limited by the fact that the children analysed are significantly older than 42 months. But the developmental profiles were a valuable aid for a pedagogical-therapeutic routine.

In general, it is evident that all standardised and non-standardised test procedures can only characterise single aspects of the children’s abilities and/or disorders. Thus, multiple, simultaneous testing procedures—as conducted in this study—most accurately reflect the abilities and limitations of the different children.

### 2.4. Pedagogical Principles

As described above, all children were extensively tested when they started kindergarten and individual support plans were created based on the results. The criteria for these examinations included self-confidence, social skills, knowledge of materials, and motor skills (Appendix C and Appendix D). The development of these abilities, which were either missing or not fully developed according to age, was then specifically supported by pedagogical means. Typically, a pedagogic review was conducted four times during the therapy year and the support measures were adjusted accordingly. As stated, pedagogical principles for the children focused on the criteria “temperament and personality functions”, “energy and drive functions”, “basic learning”, and “general tasks and demands”(ICF-CY 2007) [32].

The specialised educators (1.5 educators for 8 children) tried to improve the children’s abilities with targeted measures, entertained them, and sang with them. Reading aloud was not possible due to the children’s impaired abilities. In contrast to the speech therapists, the educators did not correct the children in their speech—linguistic corrections were made only by the speech therapists. The specialised educators and one speech therapist were available to provide long-term care for these nine children. In addition, one psychologist and one motopedist were sometimes available.

### 2.5. Video Documentation and Analysis

The individual quantitative examinations of communication behaviour were performed using video analyses of the children at the beginning of the therapy year (0 months) and after 3, 7, and 10 months. Speech, behaviour, and verbal communication skills of all children in the study group were documented for one hour, starting from 8:15 to 9:15 a.m. During this observation period, the children acted autonomously without intervention from teachers and/or therapists.

Evaluation of the recordings was conducted by a speech-language therapist, an educator specialised in speech-language therapy, and a paediatric medical expert specialising in treatment of children with language impairment. In borderline situations, the assessment of the majority of assessors was accepted.

The verbal communication behaviour of the children involved was analysed for a duration of 10 s every 2 min. The three assessors categorised the quantitative communication behaviour of the children as “verbal expressive action” (Av) and the language reaction or answer as “verbal responsive action” (Rv). In addition, “non-verbal expressive action” (An), such as hand gestures or facial expressions, “non-verbal responsive action” (Rn), and “no interaction” (D0) were evaluated. The criteria for the assessment of non-verbal responsive actions were observable gestures, movement, and significant facial expressions. Highly subtle reactions, such as a wink, were neglected due to the scope of this investigation. However, it has previously been shown that these reactions are significant in the assessment of communication skills [33].

The communication activities Av, Rv, An, Rn, and D0 were extrapolated for the observation hours and presented as percentage proportions per hour (%/h). Consequently, 28 to 30 evaluations per hour were conducted for each child, and a total of 4 observation hours were recorded at 0, 3, 7, and 10 months throughout the therapy year.

**Figure 1:**
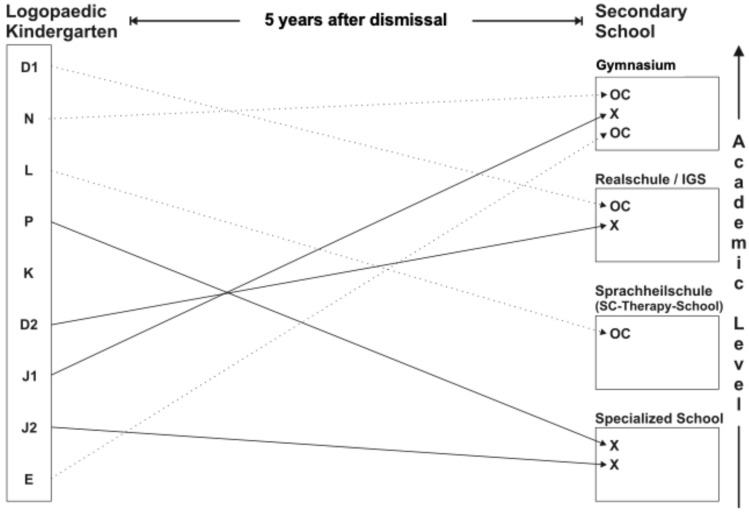
Long-term school development of the children (OC: children with predominantly communication problems: D1, N, L, E); X: children with predominantly speech-language problems: P, D2, J1, J2) (Appendix E).

In 2022, five years after discharge, a telephone follow-up survey was conducted to evaluate the study participants, including their current level of school education (Figure 1).

The primary outcome measure was the quantitative and qualitative change in interpersonal speech-language (SL) and communication behaviour in the kindergarten group (peer group) over the course of one therapy year, as measured by standardised test procedures, but also judged by subjective assessments of the therapy team. An objective method to assess communication was video documentation.

Secondary outcome measures included the level of secondary schooling achieved and language skills at the time of, and following, discharge from the kindergarten.

### 2.6. Problems Concerning Statistical Analysis

Three assessors (a speech-language therapist, an educator specialised in speech-language therapy, and a paediatric medical expert) conducted the video analysis. All three evaluators were involved in the study and were informed about the study parameter “communication”. There was consensus in almost all cases in the video assessment of the children’s communication behaviour. However, despite best efforts, experimenter bias cannot be completely ruled out.

Further statistical peculiarities: The present study is a “psychological study” with the known peculiarities and limitations of a small number of subjects. Statistical and assessment problems in psychological studies can arise, among other things, from differences between laboratory and field studies, experimenter bias, problems of generalisability, individual differences and subjective problems of the subjects, and effects of confounding factors. In addition, the effect size of the phenomenon under investigation (e.g., Cohen’s effect size) also plays a role in psychological studies, as this can, among other things, provide a measure of the significance of an observed effect. However, the statistical analysis of the various influences requires a control group or larger study groups, which is not the case in the present study. Even though no statistical data is available, the observed effect size of the parameter “communication” appears to be high in the present investigation.

### 2.7. Significance of Other Influencing Factors

The authors are aware that certain factors influencing language and communication development were not considered in this study that may alter results. Examples include the children’s IQ, the number of siblings, or similar variables. Among others, Alons et al. [16] and Cunningham et al. [8] have documented a wide range of such influencing factors.

The authors believe that, despite (or perhaps because of) the data reduction performed, the study results provide important insights into children’s development of language and communication skills.

In terms of study design, this exploratory study involves a small number of subjects, making statistical analysis mathematically inappropriate.

## 3. Results

**Figure 2:**
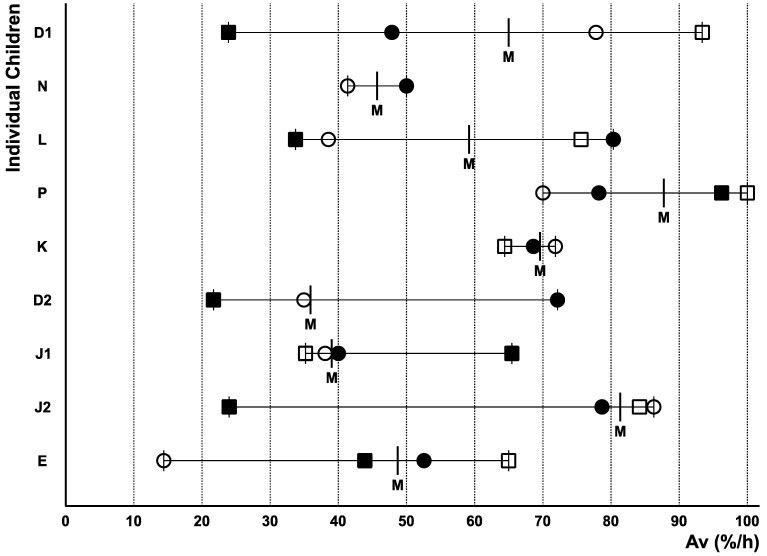
Verbal address Av in percentage per hour (%/h) of the various children at the observation times, 0 (○), 3 (●), 7 (□), and 10 (∎) months; median: (M).

All study participants demonstrated verbal communication disorders to varying degrees and with different problems (Appendix A). Figure 2 summarises verbal address (Av) and shows that all the children communicated or tried to communicate to varying degrees regardless of their individual disorders. Within the therapy year no systematic development is detectable.

The ratio between Av (active verbal expressive action) and Rv (verbal responsive action) was defined as an indicator for individual social communication (communicative participation) of the children. The authors classified the Av/Rv ratio with a range between ≤0.9 and 1.1 as “normal”, indicating an appropriate balance between verbal expression and reaction. An Av/Rv ratio of more than 1.1 due to a low Rv was classified as “irregular, less communicative”.

A general correlation was observed between expressive Av (%/h) and responsive Rv (%/h) action with a Pearson correlation coefficient of r = 0.881. The correlation between An (%/h) and Rn (%/h) for the corresponding non-verbal communication behaviour was considerably lower, with a Pearson correlation coefficient of r = 0.475.

**Figure 3:**
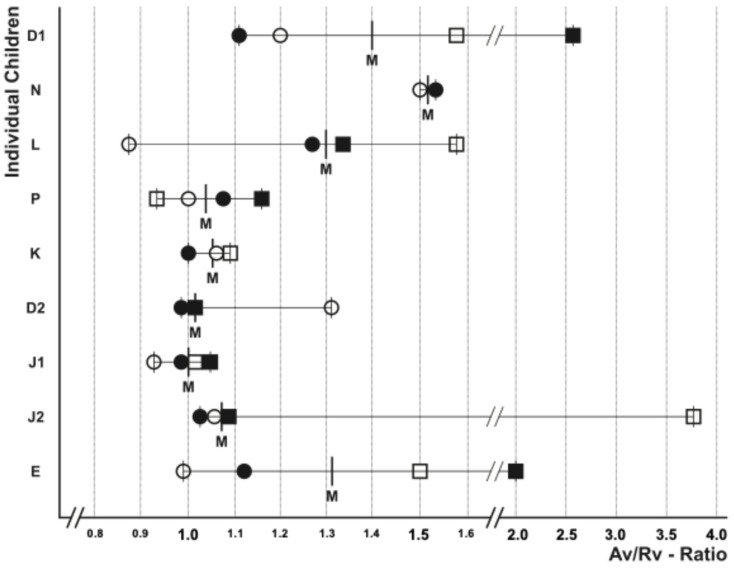
Av/Rv-ratio (verbal address/verbal response) of the various children at the observation times, 0 (○), 3 (●), 7 (□), and 10 (∎) months; median: (M).

**Figure 4:**
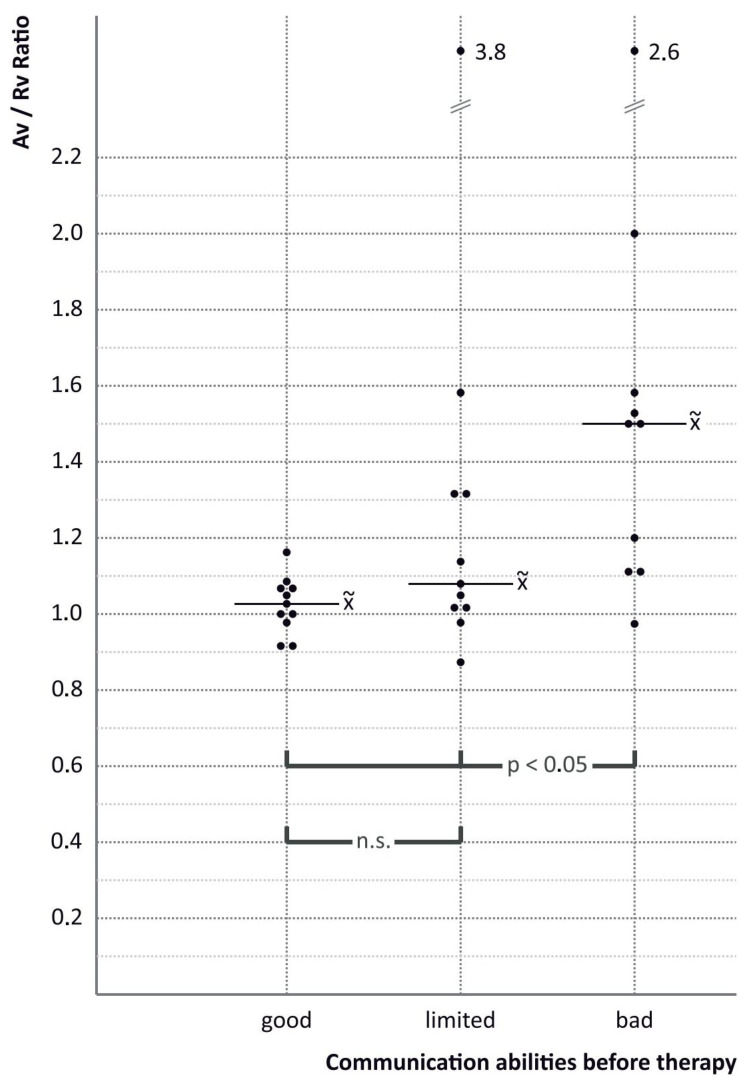
Relations between Av/Rv-ratios during therapy period and “communication abilities before therapy” (based on assessment of speech therapists and educators), with median $\tilde{x}$.

The balance between “verbal expressive action” and “responsive reaction” is thought to be important for verbal interpersonal communication. The authors expressed this via repeated evaluation of the Av/Rv ratio (i.e., the ratio of verbal expressive action to verbal response), as shown in Figure 3. This Av/Rv ratio proved to be a useful method for quantifying communication behaviour, although it varies significantly throughout the year. The median Av/Rv ratio correlated with the additional assessments of communication behaviour (Figure 4, Table 3, and Appendix A and Appendix B). During these video investigations, children acted independently of adult presence and influence, thus being able to solely communicate with their peers. It has previously been shown that the presence of adults alters communication behaviour in preschool children [3,34,35].

Figure 3 demonstrates a median Av/Rv ratio greater than 1.1 for children D1, N, L, and E. The authors interpreted this ratio as non-reciprocal communication behaviour, displaying a large amount of verbal share Av (in part without the apparent intention to communicate) and a low reactive share Rv. Children P, K, D2, J1, and J2 with an Av/Rv ratio between ≤0.9 and 1.1 appeared to have predominantly “normal” communication behaviour, with equally weighted proportions of responsive and reactive verbal behaviour, therefore displaying a “give and take” behaviour. In the present study the Av/Rv ratio fluctuated substantially over the course of the study year.

Clearly observable non-verbal communication (consisting of actions (An) and non-verbal reactions (Rn)) was rarely detected, with a median of 5% per hour (range: 2–12.5% per hour).

According to the assessment of speech therapists, educators, and test procedures in general, the children’s linguistic phonological, phonetic, and grammatical progress was better than their verbal communicative progress over the course of the therapy year (Figure 4, Table 3, Appendix A).

In a telephone follow-up five years after discharge, three (D1, N, E) of the four children (D1, N, E, L) with a high Av/Rv ratio (Av/Rv ≥ 1.1) and the assessment “pronounced communication disorder” were attending a “high-level secondary school” (German “Gymnasium”/”Realschule”/IGS; characterised by the academic level, these refer to types of secondary schools) (Figure 1; characterisation of school types in Germany: see Appendix E). In comparison, two (P, J2) of four children (P, D2, J1, J2) with significant speech-language impairment and presumed “normal” communication behaviour (based on Av/Rv < 1.1) were attending a “specialised school” with distinct support measures.

In the present study two (L, J2) of three children with DLD and oral dysfunction (L, D2, J2) had to attend a “specialised school” or “speech therapy school” in the long term.

## 4. Discussion

In this study all examined preschool children suffered from DLD and all of them expressed their desire to communicate with their peers. The verbal communication behaviour of the children as core competence for their individual development and communicative participation was determined by their peer group without adult influence. Children’s communication amongst their peers varies from that displayed in the presence of adults [3,35], in particular in young children developing language skills and children with language disorders. Research findings give some evidence that not necessarily logopaedic therapy methods, but practice and environment might be decisive for a successful development [8,11,18] similar to pedagogical development [23,24].

All children in this study showed varying degrees of improvement in linguistic aspects such as phonology, phonetics, and/or grammar during the therapy year, as determined by test procedures and the assessment of therapists. This result is in agreement with the studies of Cunningham et al. [3,35], Levickis [11], and Law et al. [36]. In contrast, unexpectedly, the examined children showed no significant changes or development in communicative verbal address (Av) or in the ratio Av/Rv as measured by objective video documentation throughout the year. Presumably, the ratio Av/Rv is an important hint for individual and interpersonal communication abilities/communicative participation. These observations were interpreted as a long-term verbal communication disorder and are consistent with the published literature [37]. According to Buzhardt et al. [38] the simultaneous impairments in speech-language and communication may be an indicator for increased risk for autism spectrum disorder. However, in the present study all children with communicative problems analysed gave no evidence for autism spectrum disorder and even showed positive speech/language and communicative development in the long term.

For the participation and development of children, linguistic abilities as well as communication skills and social abilities are highly important [1,5,39]. Whilst very often therapy differentiates between linguistic and communicative abilities, linguistic abilities are an important prerequisite for communication. Linguistic abilities can be supported and improved by intensive speech therapy [40]. For the development of communication skills and communicative participation, the authors believe that in addition to the linguistic skills, a combination of exercise and environment are crucial and that logopaedic and pedagogical support are important additional measures. This interpretation of the study findings is in concurrence with other studies [7,8,23].

In the present study, all preschool children with DLD showed a strong desire to communicate with one another, despite their speech and communication challenges. This finding shows how children use their communication abilities to engage with others and seems to indicate a strong individual intrinsic motivation [7,10,11,41,42].

Although no significant improvement in children with mainly communication skills was observed during the therapy period, the children’s long-term development seemed surprisingly successful, illustrated by their attendance at regular secondary schools. In comparison to the above group, the long-term academic development of the children with predominant DLD and “normal” communicative behaviour was less accomplished.

The observation of a positive development of communication after intensive pedagogical care during preschool age also corresponds to the observations of Mortensen et al. [43], Whitehouse et al. [44], Dickinson et al. [45], and others. It is debatable whether social, educational, or physical problems or a lack of educational support are critical determinants of the long-term problems of children with language development and communication disorders, as described by Botting et al. [46], Maggio et al. [47], and Johnson et al. [48]. From the pedagogical aspect Stamm [23,24] emphasises the importance of practice and a positive environment, which is in accordance with the findings of Cunningham et al. [8] and Alons [16].

The present study offers valuable insights to the children’s perspective to use communication for engagement in life, but also has certain limitations. It is a prospective, long-term observational study focused on the communication behaviour of preschool children with DLD in a speech therapy kindergarten. Unlike many studies that rely on questionnaires, this research aims to provide a more direct objective and comprehensive understanding through video documentation, various tests, and evaluations by specialised professionals. However, the sample size is small, with just nine children who vary significantly in their individual conditions, such as number of siblings, mother tongue, and others. The data was assessed and summarised by three examiners as described above (a speech-language therapist, an educator specialised in speech-language therapy, and a paediatric medical expert). Additional limitations include the intra-observer reliability of grading, the socio-economic context of the kindergarten studied, and potential linguistic characteristics unique to the German language.

In conclusion, although the study was limited by a small sample size and a simplified model, the findings suggest that the speech-language and communication environment plays a crucial role for the development of children. Results indicate that all children have a strong desire to engage in communicative participation with their peers. There is also some evidence that the first signs of communication problems can be recognised early in young children. In addition, we believe that video documentation and subsequent computerised analysis (for example, utilising machine learning or artificial intelligence) can also be a significant aid in the future assessment of possible communicative disorders in preschool children with DLD.

### Ethics Considerations of This Study

This study involved a secondary analysis of pseudonymised data originally collected during routine diagnostic and therapeutic speech therapy procedures in a kindergarten. No additional diagnostic interventions were conducted for research purposes, and all video recordings were deleted prior to analysis. Parental consent was obtained at admission for data use in diagnostic documentation and for specific therapeutic video recordings. No identifiable information was retained, and the research posed no additional risk to participants. The Ethics Committee of the Lower Saxony Medical Association was consulted after the study concluded and declined to issue a retrospective opinion, citing ambiguity regarding the need for prior consultation in such cases. Although no ethical violations occurred and the Ethics Committee raised no significant concerns, in future work we will seek formal ethics approval prior to the start of data collection, regardless of the perceived level of risk, to ensure alignment with best research practices.

## 5. Conclusions

This study is a prospective study in which we expected a continuous, largely parallel improvement of speech and verbal communication due to continuous therapy. However, findings show that linguistic ability of speech-language-disturbed children does not necessarily develop simultaneously with their communication ability. Evidence suggests that two groups with different disorder foci emerged, and they differed over the course of the therapy year. The present study suggests that children with mostly DLD problems predominantly require speech therapy. Children with a predominant verbal communication problem could have an incipient or existing “social (pragmatic) communication disorder”. According to the current literature, a combination of educational and speech therapy seems to be helpful for this type of disorder. Overall, our findings show that an improvement in language skills does not necessarily lead immediately to improved communication behaviour.

Despite the aforementioned limitations and the small, heterogeneous group of children with DLD studied—along with the resulting statistical challenges—the authors believe that further large-scale studies would be beneficial.

### What This Paper Adds

Little is known about the relations between language and verbal communication development in preschool children with DLD. In general, it is assumed that language and verbal communication development occur largely in parallel.

In the present study, evidence suggests that young children can be divided into two distinct groups: one group primarily exhibits language disorders, while the other shows predominantly verbal communication difficulties. However, independent of their impairment, all children want to engage in communication/communicative participation with their peers. It is important to note that speech-language therapy may not lead to simultaneous improvements in speech-language and communication skills.

The authors suggest that speech therapy should be sought for preschool children with a speech disorder, and a combination of educational and speech therapy interventions should be used for children with a verbal communication disorder.

## Figures and Tables

**Table 1:** Characterisation of the children.

Child	Age (y) *1	Native Family Language	IQ (SON-R) *2 [26]	Number of Siblings	ICD-10 *3 [25]
D1	6	Polish	h	1	F80-9
N	5	German	a	1	F80.9
L	5	German	a	1	F80.9, F82.9
P	5	Russian	a(b), borderline	0	F80.9
K	5	German	a	1	F80.9
D2	5	Polish	a	1	F80.9, F82.9
J1	5	Russian	h	9	F80.9
J2	5	German	a	1	F80.9, F82.9
E	4	Russian	a	1	F80.9

*1: Beginning of the study; *2: a (average; 90–110), b (below average, <90), h (higher than average, >110); *3: International Classification of Diseases.

**Table 2:** Test procedures in the logopaedic kindergarten.

Skills	Test Procedure	Validated
Language + Speech	SETK—3–5 Test for language/speech development of children 3–5 years [27]	yes
	AWST-R—Vocabulary test for children 3–5 years [28]	yes
	PDSS—Patholinguistic diagnostics for speech-language-impaired children [29]	yes
Cognition	Kaufmann Assessment Battery for Children II (KABC-II 2015) [30]	yes
	SON-R2.5–7—Non-verbal IQ test for children 2.5–7 years [26]	yes
Motor skills + Movement	MOT 4–6—Test for children 4–6 years [31]	yes

**Table 3:** Linguistic abilities at the beginning and the end of the therapy year based on logopaedic and educational assessment.

Child	Linguistic Skills Before Therapy	Linguistic Skills After Therapy
D1	hardly any dialogue ability	partially good dialogue ability
N	hardly any dialogue ability	good dialogue ability
L	limited dialogue ability	good dialogue ability
P	good dialogue ability	good dialogue ability
K	good dialogue ability	good dialogue ability
D2	limited dialogue ability	good dialogue ability
J1	limited dialogue ability	good dialogue ability
J2	limited dialogue ability	good dialogue ability
E	hardly any dialogue ability	good dialogue ability

## Data Availability

The original contributions presented in this study are included in the article. Further inquiries can be directed to the corresponding author(s).

## Appendix A

**Table A1:** Speech-language development status at the beginning (B) and end (E) of speech therapy in the logopaedic kindergarten (based on assessment by SL teachers).

	D1 B E	N B E	L B E	P B E	K B E	D2 B E	J1 B E	J2 B E	E B E
Articulation	6 4	5 3	4 3	4 3	4 3	4 3	5 2	5 4	4 4
Orofacial complex	4 4	4 2	3 2	4 2	3 3	3 2	3 2	4 3	1 1
Semantic lexical	5 5	4 3	3 1	4 3	4 3	5 3	4 1	5 1	4 4
Grammar Syntax	6 4	6 2	4 2	4 3	5 3	5 3	4 2	4 3	5 4
Grammar Morphology	6 4	6 4	4 3	4 3	3 3	4 3	4 3	4 3	6 4
Language understanding	5 4	4 2	2 1	4 3	4 3	3 2	3 1	3 1	5 3
Auditory Processing	6 3	5 3	5 3	4 3	3 3	4 3	3 3	4 2	4 3
Communicative pragmatic	6 4	5 2	4 2	3 3	4 3	5 3	4 3	4 3	5 2

1: age-appropriate, trouble-free; 2: slightly conspicuous; 3: partially, still defective; 4: defective, multiple; limited; 5: considerably disturbed, severe; 6: not assessable, not present.

## Appendix B

**Table A2:** Skills necessary for speech-language development before and after therapy (according to Zollinger [15]. The data refer to individual abilities compared to normally developed 3.5-year-old children. Legend: (↓)↓: (strongly) impaired; Ø: average; ↑: enhanced.

Name	IQ	Before Therapy				Age (Years)	After Therapy			
		Practical-Gnostic	Symbolic	Linguistic	Social Communicative		Social Communicative	Linguistic	Symbolic	Practical- Gnostic
D1	↑	↓↓	Ø	↓↓	↓↓	6	↓↓	Ø	Ø	Ø
N	Ø	Ø	Ø	↓↓	↓↓	5	↓↓	↓↓	Ø	Ø
L	Ø	Ø	Ø	↓↓	↓	5	Ø	↓↓	Ø	Ø
P	↓	Ø	Ø	↓↓	↓↓	5	↓↓	Ø	Ø	Ø
K	Ø	Ø	Ø	↓	Ø	5	Ø	↓	Ø	Ø
D2		Ø	Ø	↓↓	↓↓	5	↓↓	↓↓	Ø	Ø
J1	↑	Ø	Ø	Ø	Ø	5	Ø	Ø	Ø	Ø
J2		Ø	Ø	↓↓	↓	5	Ø	Ø	Ø	Ø
E	Ø	Ø	↓	↓	Ø	4	Ø	Ø	↓Ø	Ø

## Appendix C

**Table A3:** Non-verbal parameter for the assessment of emotional and social qualities (assessed by educators).

Parameters	Assessment Score		
	Below Average	Average	Above Average
Frustration tolerance	1	2	3
Ability to accept criticism	1	2	3
Social competence	1	2	3
Compliance with rules	1	2	3
Ability to form relationships	1	2	3
Maximum points			15
Average points		10	
Minimum points	5		

## Appendix D

**Table A4:** Assessment of social and emotional qualities of the various children by educators and therapists. Criteria as shown in Table A3.

	Child								
	D1	N	L	P	K	D2	J1	J2	E
Frustration tolerance	1	2	2	1	2	1	2	3	1
Ability to accept criticism	2	2	2	2	2	2	3	2	1
Social competence	2	3	2	1	2	3	3	3	2
Compliance with rules	3	3	2	1	2	3	3	1	2
Ability to form relationships	1	1	3	3	2	2	1	2	1
Total Points	9	11	11	8	10	11	12	11	7

## Appendix E. Characterisation of School Types in Germany

The German “Gymnasium” specifically refers to a type of secondary high school that prepares students for university education.

“Realschule” refers to a type of secondary school that provides a more practical and vocational education compared to the academically focused Gymnasium, typically for students aged around 10 to 16.

The “IGS” integrates various educational tracks, offering a range of academic and vocational courses within the same institution, allowing students to choose different pathways based on their abilities and interests.
